# Guideline-directed medical therapy in older adults with heart failure; Are there differences across age group?

**DOI:** 10.1186/s12877-026-07571-y

**Published:** 2026-05-07

**Authors:** Schee Li Kwa, Bui Khiong Chung, Zhun Foo Tan, Janice Kee Wei Wong, Zi Yang Lian, Kok Choon Cheah, Ka-Shing Tan, Chun-Hau Lam, Mae Jane Khaw, Siew Yap Chai, Melvinder Singh A/L Jasbinder, Lee Yee Ting, Jordan Ching Bing Hoo, Chelfi Zhi Fei Chua, Min Ting Wong, Pey Woei Ting, Jun Xun Ng, Qing Wei Lim, Ching Hua Chai, Wei Ming Cho, Alex Zhi Yang Koh, Tze Cheng Wong, Weng Kee Ho, Wun Khang Chong, Rebecca Su Li Chew, Jothiswaran Namasoo, Ing Chiew Yew, Hwei Sung Ling

**Affiliations:** 1 Department of Medicine, Sri Aman Hospital, Sarawak, Malaysia; 2 Department of Cardiology, Sarawak Heart Centre, Sarawak, Malaysia; 3 Faculty of Medicine and Health Sciences, Universiti Malaysia Sarawak, Kota Samarahan, Malaysia; 4 Department of Medicine, Sarikei Hospital, Sarawak, Malaysia; 5 Department of Medicine, Limbang Hospital, Sarawak, Malaysia; 6 Department of Medicine, Kapit Hospital, Sarawak, Malaysia; 7 Department of Medicine, Serian Hospital, Sarawak, Malaysia; 8 Department of Pharmacy Sarawak Heart Centre, Sarawak, Malaysia; 9 Department of Medicine, Bintulu Hospital, Sarawak, Malaysia; 10 Department of Medicine, Miri Hospital, Sarawak, Malaysia; 11 Department of Medicine, Sarawak General Hospital, Sarawak, Malaysia; 12 Department of Medicine, Sibu Hospital, Sarawak, Malaysia

**Keywords:** Heart failure, Guideline-directed medical therapy, Older adults/ Geriatric, Mortality and heart failure readmissions, Age stratification

## Abstract

**Title:**

Guideline-Directed Medical Therapy in Older Adults with Heart Failure; Are There Differences Across Age Groups?

**Background:**

Older adults remain underrepresented in heart failure (HF) studies evaluating guideline-directed medical therapy (GDMT). This study aimed to assess clinical characteristics, HF GDMT pillars use, and outcomes across age groups of older adults with HF.

**Methods:**

This retrospective, multicentre sub-analysis of the Sarawak Heart Failure Registry included patients aged ≥ 65 years. Participants were stratified into youngest-old (65–70 years), middle-old (71–75 years), and oldest-old (> 75 years). GDMT use, dosing, and clinical outcomes, including all-cause mortality and HF readmission, were evaluated at baseline, 3 and 6 months. Multivariable regression analyses were performed to identify factors associated with outcomes and GDMT optimisation.

**Results:**

A total of 176 patients were included. Comorbidity burden increased with age, whereas coronary artery disease was more prevalent in the youngest-old group. HF GDMT pillars use declined with increasing age, particularly for renin–angiotensin system inhibitors and SGLT2 inhibitors, while beta-blocker use remained consistently high. At 6 months, 50% of the oldest-old achieved three GDMT pillars, although fewer achieved four compared with younger groups. LVEF improved in all age groups, most notably in the youngest-old. NYHA functional class improvement was greater in younger patients. Mortality at 6 months was numerically higher in the youngest-old (16.0%), while HF readmission was slightly higher in the oldest-old (12.5%), without statistical significance. In multivariable analyses, age, comorbidities, and LVEF were not independent predictors of outcomes.

**Conclusion:**

Older adults with HF demonstrated consistent structural and functional improvement across age groups despite differences in GDMT use. Outcome variation was not independently explained by age or comorbidity burden, suggesting that disease phenotype and physiological reserve may play a greater role. These findings provide real-world evidence from a multiethnic Asian population and support individualized, multidisciplinary care strategies for older adults with HF.

**Supplementary Information:**

The online version contains supplementary material available at 10.1186/s12877-026-07571-y.

## Background

The global ageing population is increasing, particularly in the Asia-Pacific region. In Malaysia, the older adults population is growing rapidly, with projections indicating it will double by 2050 [1]. Currently, Malaysia’s life expectancy stands at 75 years [1]. In older adults, similar to their younger counterparts, the pathophysiology of heart failure is characterized by systemic congestion and neurohormonal dysregulation. Diuretics relieve congestion, while the HF guideline-directed medical therapy (GDMT) pillars target maladaptive neurohormonal activation [2]. HF GDMT pillars including renin-angiotensin system inhibitors (RASi), beta-blockers (BB), mineralocorticoid receptor antagonists (MRA), and sodium-glucose cotransporter-2 inhibitors (SGLT2i) improve survival in patients with heart failure with reduced ejection fraction (HFrEF) [3–6]. However, older adults are often underrepresented in these trials. PREDICT trial found that 25.5% of 251 trials investigating heart failure treatments excluded participants based on arbitrary upper age limits [2]. Additionally, factors like comorbidities, cognitive impairment, physical disabilities, polypharmacy, and communication barriers further limit the inclusion of older patients in clinical trials [2].

In Malaysia, studies from institutions like Sarawak General Hospital Heart Failure Registry (SGH HF) [7], National Heart Institute Acute Decompensated Heart Failure Registry (IJN ADHF) [8] and Malaysian Heart Failure Registry (MYHF) [9] report that 50%-60% of heart failure patients are aged 60 or older, highlighting the significant burden of heart failure in this population. SGH-HF also shows that the regional prescription is low, emphasizing the need to examine HF GDMT pillars usage in every population [7]. Sub-analyses of the Chronic Heart failure ESC guideline-based Cardiology practice Quality project (CHECK-HF) study in the Netherlands [10] and the Korea Acute Heart Failure (KorAHF) registry [11] are among the few studies worldwide that have explored HF GDMT pillars in older adults with heart failure which showed that the rate of GDMT pillars decreased with age, with older adults being less likely to receive optimal treatment compared to younger individuals. However, the KorAHF registry [11] found that even in octogenarians, HF GDMT pillars was associated with a reduction in all-cause mortality. Older adults with heart failure often face worse outcomes due to multimorbidity, functional and cognitive impairments, and high readmission rates [12, 13]. However, whether HF GDMT pillars is equally effective in improving survival among older adults with heart failure in Malaysia remains unclear, as there is limited data on this subgroup. This study aims to explore the clinical characteristics, HF GDMT pillars prescription patterns, and outcomes of older adults with heart failure who follow up at heart failure clinics.

## Methods

### Study design

This was a sub-analysis of data collected in the Sarawak Heart Failure Registry (Sarawak HF), a retrospective, multicentre and observational study on heart failure patients.

### Sarawak heart failure registry (Sarawak HF)

The Sarawak HF registry included 576 patients treated in heart failure clinics across ten different hospitals in the state of Sarawak, between 1 January 2021 and 30 June 2023. These hospitals include Sarawak Heart Centre, Sarawak General Hospital, Sibu Hospital, Miri Hospital, Sarikei Hospital, Sri Aman Hospital, Sri Aman Hospital, Serian Hospital, Bintulu Hospital, Limbang Hospital, and Kapit Hospital.

Patients were included in the registry if they were aged > 18 years old and had heart failure diagnosis established according to the Malaysia National Heart Failure guideline. Patient with missing echocardiographic parameters and coronary anatomy imaging study were excluded.

The registry collected baseline clinical characteristics, including age, sex, ethnicity, and comorbidities such as hypertension, diabetes mellitus, coronary artery disease, atrial fibrillation, chronic kidney disease, cerebrovascular disease, obstructive airway disease, and smoking status. Baseline clinical measurements including blood pressure, body mass index (BMI), serum potassium and creatinine levels, and left ventricular ejection fraction (LVEF) were recorded.

The registry also documented the prescription patterns of HF GDMT pillars at 3-month and 6-month follow-up. Clinical outcomes including all-cause mortality, heart failure readmission at 3 and 6-month follow-up, and changes in New York Heart Association (NYHA) functional class during follow-up were recorded.

### Study settings

Sarawak, a Malaysian state located on the island of Borneo, has two centres with geriatric services and one cardiology centre within the public healthcare system. Heart failure care at the cardiology centre is delivered by cardiologists, whereas in district hospitals without subspecialty services, general physicians provide both geriatric care in general clinics and heart failure management through dedicated heart failure clinics. Consequently, HF management across centres is not protocolised but is guided by contemporary clinical practice guideline recommendations, with the aim of initiating and optimising HF GDMT pillars as tolerated. Initiation and up-titration of therapy were accompanied by routine monitoring of blood pressure, renal function, and serum potassium. Although clinic visit frequency varied between institutions and was influenced by local resource constraints, all centres prioritised timely optimisation of HF GDMT pillars to the maximally tolerated dose.

### Patient population, variables and clinical outcomes for the current sub-analysis

For the current sub-analysis of the above Sarawak HF registry, all older patients aged ≥ 65 years were included. This cohort were stratified into 3 groups: Youngest Old (65–70 years old); Middle Old (71–75 years old); Oldest Old (> 75 years old), following the current life expectancy in Malaysia which is around 75 years old [1].

Sub-analysis performed comparing 3 groups at baseline, 3-month and 6-month follow up were: baseline clinical characteristics (demographics and comorbidities); HF GDMT pillars prescription patterns (the individual HF GDMT pillars prescriptions, number of types of HF GDMT pillars prescribed, achieving at least 50% target dose); NYHA functional class; mean LVEF and clinical outcomes (all-cause mortality, HF readmission). We included all baseline clinical characteristics variables from the earlier Sarawak HF registry. Our analysis grouped angiotensin converting enzyme (ACEi), angiotensin receptor blocker (ARB) and angiotensin neprilysin receptor blocker (ARNi) under the renin-angiotensin system inhibitors (RASi) group. Other HF GDMT pillars include beta blockers (BB), mineralocorticoid receptor antagonists (MRA) and sodium-glucose contransporter-2 inhibitor (SGLT2i).

#### Definitions

1. The rationale of age group stratification was based on the past geriatric research on the functional differences throughout stages of aging and Malaysia life expectancy of 75 years old [1, 14].
2. Renal function was classified based on estimated glomerular filtration rate (eGFR) [15]. The eGFR was calculated using the CKD Epidemiology Collaboration creatinine Eq. (2021).
3. Patients were classified according to baseline left-ventricular ejection fraction (LVEF) into HF with reduced ejection fraction (HFrEF; LVEF < 40%), HF with mildly reduced ejection fraction (HFmrEF; LVEF = 40–49%) and HF with preserved ejection fraction (HFpEF; LVEF ≥ 50%) [16].

### Statistical analysis

Categorical variables were expressed as frequencies (n) and percentages (%) and compared using the chi-square test or Fisher’s exact test where appropriate. Fisher’s exact test was applied when more than 20% of cells had expected counts less than 5. Continuous variables were presented as mean ± standard deviation (SD) for normally distributed data or median with interquartile range (IQR) for non-normally distributed data. When a statistically significant difference was detected, post-hoc pairwise comparisons between age groups were performed using Bonferroni correction to adjust for multiple testing.

All-cause mortality and heart failure readmission at 3 and 6 months, were compared using the chi-square test or Fisher’s exact test where appropriate, and relative risks (RRs) with 95% confidence intervals (CIs) were additionally reported.

Multivariable logistic regression analyses were performed to identify factors associated with all-cause mortality and heart failure readmission at 3 and 6 months. Multivariable ordinal logistic regression analysis was used to assess factors associated with the number of HF GDMT pillars prescribed at 3 and 6 months. Covariates significantly different between age groups and known predictors of heart failure outcomes were included [17]. All analyses were performed using IBM SPSS Statistics version 28.0.1.0 (IBM Corp., Armonk, NY, USA), and a two-sided p value < 0.05 was considered statistically significant.

### Human ethics and consent to participate

This report presents findings from a subset of participants enrolled in an observational heart failure registry. The main study, titled *Evaluation of Heart Failure Management and Outcome in Heart Failure Clinics in Sarawak*, was registered with the National Medical Research Register and received approval from the Malaysian Research Ethics Committee (NMRR-23-01606-FPD). The investigation conforms with the principles outlined in the Declaration of Helsinki. Written informed consent was obtained from all participants prior to their inclusion in the heart failure registry.

## Result

### Baseline clinical characteristics

Among 578 patients in the Sarawak HF registry, 176 (30.4%) were aged ≥ 65 years and included in this analysis. These were stratified into youngest-old (65–70 years, *n* = 86, 48.9%), middle-old (71–75 years, *n* = 49, 27.8%), and oldest-old (> 75 years, *n* = 41, 23.3%).

As summarised in Table 1, the proportion of male patients decreased with age (76.7% vs. 59.2% vs. 65.3%, *p* = 0.005), while the prevalence of chronic kidney disease increased (32.4% vs. 45.2% vs. 60.0%, *p* = 0.025). Other comorbidities were comparable across groups, although the oldest-old group showed numerically higher rates of hypertension, diabetes mellitus, atrial fibrillation, and stroke. In contrast, coronary artery disease was more prevalent in the youngest-old group.

**Table 1:** Baseline clinical characteristics in older adults with heart failure

	Age group (*n* = 176)						
	65–70 Youngest-old (*n* = 86)		71–75 Middle-old (*n* = 49)		> 75 Oldest-old (*n* = 41)		
	Mean/ *n *	SD/ (%)	Mean	SD/ (%)	Mean	SD/ (%)	*p* value
Age	67.0	1.7	73.0	1.3	79.0	3.7	< 0.001*
Male gender	66	(76.7)	29	(59.2)	20	(65.3)	0.005*
BMI, kg/m^2^	24.7	4.4	22.8	4.9	22.8	3.8	0.079
Race							
Malay	31	(36.5)	9	(18.4)	10	(25)	0.223
Chinese	20	(23.5)	13	(26.5)	13	(32.5)	
Indian	1	(1.2)	0	0	0	0	
Others Ethnic	33	(38.8)	27	(55.1)	17	(42.5)	
Smokers	32	(37.2)	12	(24.5)	11	(26.8)	0.031*
LVEF, %	29.0	9.7	35.0	14.7	36.0	12.4	0.001*
EF group							
HFpEF	3.0	(3.6)	7	(14.3)	6	(15.4)	0.019*
HFmrEF	3	(3.6)	5	(10.2)	4	(10.3)	
HFrEF	78	(92.9)	37	(75.5)	29	(74.4)	
NYHA							
I	21	(26.9)	8	(18.6)	4	(13.8)	0.325
II	43	(55.1)	22	(51.2)	17	(58.6)	
III	12	(15.4)	13	(30.2)	8	(27.6)	
IV	2	(2.6)	0	0	0	0	
History HF admission	42	(48.8)	31	(63.3)	28	(70)	0.053
Comorbidities							
Hypertension	62	(72.1)	39	(79.6)	33	(82.5)	0.384
Diabetes Mellitus	41	(47.7)	21	(42.9)	21	(52.5)	0.666
Dyslipidaemia	52	(60.5)	33	(67.3)	27	(65.9)	0.692
Ischemic Heart Disease	49	(57)	27	(55.1)	18	(43.9)	0.387
Atrial fibrillation/ Flutter	25	(29.4)	19	(39.6)	19	(46.3)	0.157
Stroke	9	(10.5)	4	(8.3)	5	(12.2)	0.855
Obstructive Airway Disease	8	(9.3)	11	(7.1)	7	(17.1)	0.089
Myocardial Infarction	21	(24.4)	8	(16.7)	3	(7.3)	0.060
Valvular Heart Disease	9	(10.5)	7	(14.9)	6	(14.6)	0.724
CKD (eGFR<60 ml/min)	23	(32.4)	19	(45.2)	21	(60)	0.025*
CKD stage							
1	16	(22.5)	6	(14.3)	2	(5.7)	0.029*
2	32	(45.1)	17	(40.5)	12	(34.3)	
3	18	(25.4)	18	(42.9)	15	(42.9)	
4	3	(4.2)	1	(2.4)	6	(17.1)	
5	2	(2.8)	0	0	0	0	
Systolic BP, mmHg	123.0	19.0	133.0	26.6	134.0	22.9	0.025*
Diastolic BP, mmHg	74.0	12.9	75.0	15.1	76.0	12.1	0.861
Heart rate, bpm	81.0	20.0	80.0	19.0	82.0	15.0	0.905

*SD* Standard Deviation, *BMI* Body Mass Index, *LVEF* Left ventricular Ejection Function, *HFrEF* Heart failure reduced ejection fraction, *HFmrEF* Heart Failure mildly reduced ejection fraction, *HFpEF* Heart Failure preserve ejection fraction, *NYHA* New York Heart Association classification, *CKD* Chronic kidney disease

*p value is significant

HFrEF was the predominant phenotype across all age groups, with the highest proportion in the youngest-old, whereas HFpEF was more common in the oldest-old (*p* = 0.019). Mean LVEF increased with age (Table 1). Most patients were classified as NYHA class II at baseline, although a higher proportion of class III was observed in the middle-old and oldest-old groups.

### HF GDMT pillars

**Fig. 1:**
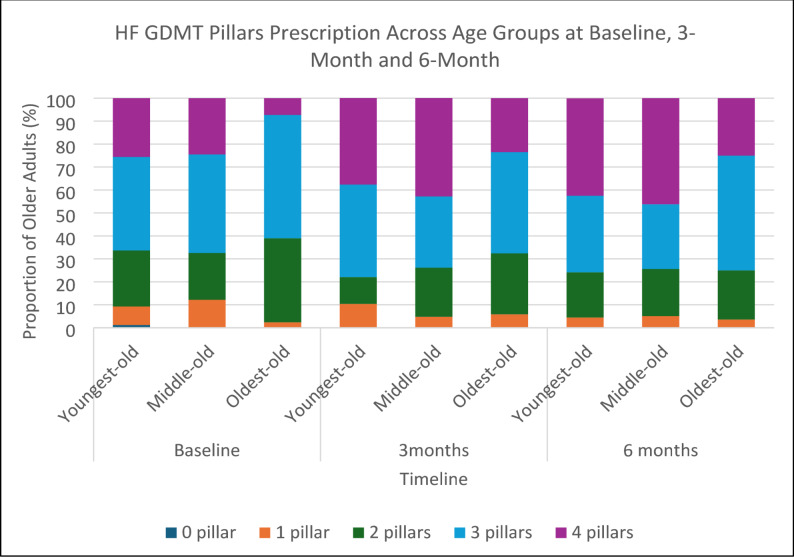
Number of HF GDMT pillars across age groups at baseline, 3-month and 6-month

The number of HF GDMT pillar prescription across age groups is summarized in Fig. 1 and supplementary Table 1. At baseline, most patients received three GDMT pillars, while fewer achieved four, particularly in the oldest-old group (7.3% vs. 25.6% and 24.5%). Following optimization, the proportion achieving four pillars increased at 3 and 6 months, particularly in the younger groups. At 6 months, 42.4% of the youngest-old and 46.2% of the middle-old groups achieved four pillars, compared with 25.0% in the oldest-old group, although half of the oldest-old achieved three pillars.

Individual HF GDMT prescriptions are shown in Supplementary Table 2. RAS inhibitor (Supplementary Fig. 2a) use was lowest in the oldest-old group, with a significant difference at 3 months (64.7% vs. 87.0% and 83.3%, *p* = 0.020), but similar rates across groups at 6 months. Beta-blocker (Supplementary Fig. 2b) use remained consistently high (> 88% at 3 months and > 90% at 6 months) without significant differences. MRA prescription (Supplementary Fig. 2c) was comparable across groups at all time points. SGLT2 inhibitor (Supplementary Fig. 2d) use was lower overall but increased modestly during follow-up, with no significant between-group differences.

At both 3 and 6 months, similar proportions of patients across age groups achieved ≥ 50% target dosing of HF GDMT therapies (Supplementary Table 2).

### NYHA assessment, LVEF and HF clinical outcomes

**Fig. 2:**
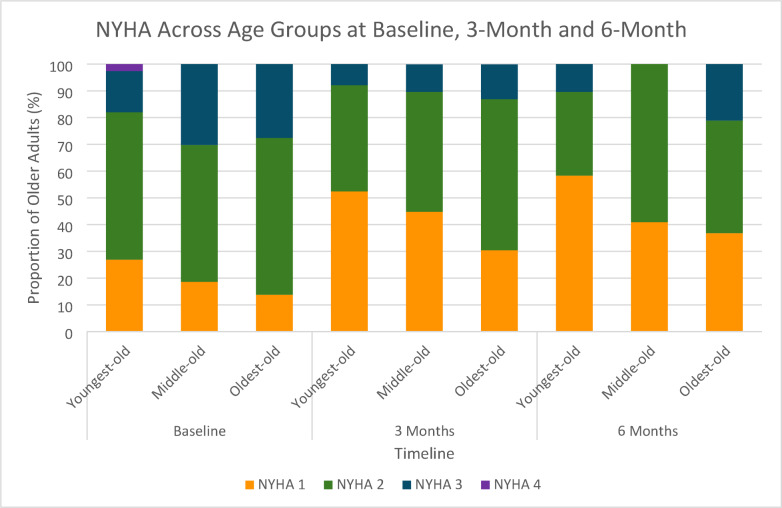
NYHA functional class across age groups at baseline, 3-month, 6-month

Changes in NYHA functional class are summarised in Fig. 2 and Supplementary Table 3. At baseline, most patients were in NYHA class II. Functional status improved across all groups during follow-up, with the greatest improvement observed in the youngest-old group, where the majority achieved NYHA class I by 3 and 6 months. In contrast, middle-old and oldest-old groups remained predominantly in NYHA class I–II.

**Fig. 3:**
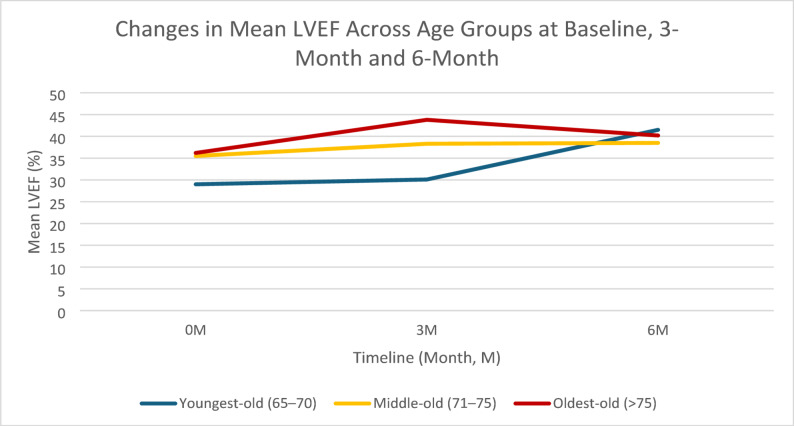
: Mean LV ejection fraction (EF) across age groups at baseline, 3-month and 6-month

LVEF trends are shown in Fig. 3 and Supplementary Table 4. At baseline, LVEF was significantly lower in the youngest-old group compared with the middle-old and oldest-old groups (29.0 ± 9.7% vs. 35.5 ± 14.7% vs. 36.2 ± 12.3%, *p* = 0.001). Post-hoc Bonferroni analysis confirmed these differences. LVEF improved across all groups during follow-up, with the greatest increase observed in the youngest-old group, although between-group differences were no longer significant at 3 and 6 months.

Clinical outcomes are summarised in Table 2. Mortality rates appeared numerically higher in the youngest-old group compared with the middle-old and oldest-old groups at both 3 months (9.8% vs. 4.3% and 5.3%) and 6 months (16.0% vs. 11.4% and 12.9%). Relative risk estimates suggested lower mortality risk in the middle-old and oldest-old groups compared with the youngest-old group; however, these differences were not statistically significant, with all confidence intervals crossing unity.

**Table 2:** All-cause mortality and heart failure readmission across age groups at 3-month and 6-month

	65–70 Youngest-old (*n* = 86)		71–75 Middle-old (*n* = 49)		> 75 Oldest-old (*n* = 41)		
	*n* (%)	RR/ 95% CI	*n* (%)	RR/ 95% CI	*n* (%)	RR/ 95% CI	*p* value
All-cause mortality							
3-month	8 (9.8)	1/ (-)	2 (4.3)	0.4/ (0.1-2.0)	2 (5.3)	0.45/ (0.1–2.5)	0.540
6-month	12 (16.0)	1/ (-)	5 (11.4)	0.6/ (0.2-2.0)	4 (12.9)	0.7/ (0.2–2.6)	0.766
HF Readmission							
3-month	6 (7.3)	1/ (-)	4 (8.7)	1.2/ (0.3–4.5)	4 (10.3)	1.4/ (0.3–5.4)	0.821
6-month	6 (8.0)	1/ (-)	6 (13.6)	1.8/ (0.5-6.0)	4 (12.5)	1.6/ (0.4–6.2)	0.507

*RR* Relative Risk, 95% *CI*, 95% Confidence Interval

*p value is significant

In contrast, heart failure readmission rates were broadly comparable across the three age groups at both follow-up time points. Although slightly higher readmission proportions were observed in the older groups, the corresponding relative risks were not statistically significant.

Overall, no significant differences in mortality or heart failure readmission were observed between age groups during follow-up.

Multivariable logistic regression (Supplementary Table 5) showed that age group was not independently associated with mortality or heart failure readmission at 3 or 6 months. Most covariates were also not significantly associated with outcomes, except hypertension, which was associated with higher odds of 6-month readmission.

Ordinal logistic regression (Supplementary Table 5) demonstrated that age group was not independently associated with HF GDMT optimisation. Male sex was associated with higher odds of receiving a greater number of HF GDMT pillars, while diabetes mellitus was associated with lower odds at 3 months and showed a borderline association at 6 months. Other variables were not significantly associated.

## Discussion

The main findings in this study were threefold. First, the use of renin–angiotensin system inhibitors (RASi), achievement of all four HF GDMT pillars, and attainment of ≥ 50% target doses were consistently lowest in the oldest-old group throughout follow-up. Second, despite overall improvement in left ventricular ejection fraction (LVEF) across all age groups, the oldest-old group showed persistently lower rates of symptomatic improvement. Third, although not statistically significant, mortality was numerically higher in the youngest-old group, whereas heart failure readmission was more frequent in the oldest-old group.

### Baseline clinical characteristics

Our study demonstrated a reduction in the number of patients with increasing age, a phenomenon also observed in the IJN ADHF [8] study in Malaysia (30.9% aged 60–69; 21.6% aged 70–79; 7.1% aged > 80). In contrast, a higher proportion of patients aged > 75 years (50%) was reported in the CHECK-HF registry [11]. This difference may reflect Malaysia’s population pyramid, which shows a smaller proportion of individuals in the oldest age groups due to natural attrition [1]. Differences in cultural beliefs [18], health literacy [19], and access to social support [20] may also influence healthcare-seeking behaviour, potentially contributing to the underrepresentation of the oldest-old in clinical settings.

Female predominance increased with advancing age, and the oldest-old group had a higher proportion of HFpEF, whereas male sex and HFrEF were more common in the youngest-old group. Similar trends were reported in octogenarians in the Euro Heart Failure Survey I [21] and the 2017 AHA Heart Disease and Stroke Statistics Update [22]. The predominance of HFpEF in the oldest-old may be explained by a higher prevalence of comorbidities such as atrial fibrillation, hypertension, and cerebrovascular disease, while women are more likely to develop diastolic dysfunction with preserved systolic function [23–25].

In our study, a higher proportion of Chinese patients with heart failure were observed in the oldest-old group compared with Malays. Previous studies from Hong Kong [26] and Toronto [27] have also reported a higher prevalence of HFpEF among Chinese patients. Hypertension, a major risk factor for HFpEF, has been shown to be more common among Chinese populations [23, 28, 29], which may partly explain the greater proportion of Chinese patients in the oldest-old group in our study.

The youngest-old group had a higher prevalence of smoking and coronary artery disease, which may contribute to the predominance of HFrEF in this group. Smilowitz et al. similarly reported that coronary artery disease is more common in the youngest-old population [30]. Although CAD generally increases with age, the higher prevalence observed in the youngest-old group in our study may reflect earlier exposure to cardiovascular risk factors such as smoking, obesity, and family history of heart disease, which accelerate the development of CAD [31].

### Guideline directed medical therapy

Current AHA/ACC and ESC guidelines recommend that elderly patients with HFrEF receive the same four pillars of GDMT—RAS inhibitors/ARNI, β-blockers, MRAs, and SGLT2 inhibitors—while considering tolerability, comorbidities, and frailty [32, 33]. In our study. HF GDMT pillars optimisation was lower in the oldest-old group than in the younger age groups, although half of the patients still achieved three HF GDMT pillars after 6 months of HF clinic follow-up. Compared with previous reports from the CHECK-HF sub-analysis and other elderly heart failure cohorts, our study demonstrated a higher proportion of oldest-old patients achieving three HF GDMT pillars [11, 34]. This difference may reflect the structured follow-up in a specialised HF clinic, including more frequent visits and proactive physician titration of GDMT to the maximally tolerated doses [35, 36], although differences in study design, patient selection, and baseline characteristics may also contribute.

RASi prescription and continuation were lowest in the oldest-old group from baseline to 6 months compared with the younger age groups. Nevertheless, overall RASi use among patients aged > 75 years in our cohort was comparable to rates reported in the CHECK-HF study and another study cohort [11, 34]. The lower titration rates observed in the oldest-old may reflect the higher prevalence of advanced chronic kidney disease (CKD) in this group. However, evidence suggests that discontinuation of RASi in advanced CKD does not improve renal outcomes and that dose reduction, rather than discontinuation after heart failure hospitalization, may preserve therapeutic benefit [37]. Thus, despite renal impairment limiting up-titration, cautious dose adjustment with monitoring may permit continued RASi therapy in selected older adults.

SGLT2i prescription was low across all age groups, likely reflecting limited drug availability in the hospital. Clinical inertia in initiating SGLT2i in older adults with multiple comorbidities and CKD may also contribute, as suggested in previous studies [38]. The benefits of SGLT2i in selected older adults with CKD should therefore be emphasized [39].

Our study demonstrated high initial MRA prescription rates among older adults with heart failure, although dose optimisation remained challenging, particularly in the oldest-old group. At 6 months, MRA prescription in the oldest-old remained higher than rates reported in Barry et al.15 and the CHECK-HF study [11], with over half achieving at least 50% of target doses. These findings highlight the complexities of managing frailty, multimorbidity, renal impairment, and polypharmacy in older patients. Recent trials of non-steroidal MRA have shown improved heart failure outcomes with favourable tolerability profiles, including among older adults [40, 41]. Despite this, MRAs remain underutilised due to concerns regarding hyperkalaemia and renal dysfunction. However, these risks may be mitigated through cautious initiation, gradual titration, and close monitoring of renal function and potassium levels [42]. Together with evidence from recent trials, our findings support the safe use of MRA in older patients with appropriate monitoring, while newer non-steroidal MRAs may further improve tolerability.

Randomised controlled trials have demonstrated that beta-blockers reduce hospitalisation and mortality in heart failure [5]. In our study, beta-blocker prescription at 6 months exceeded 90% across all age groups, including the oldest-old, which is higher than rates reported in Hamaguchi et al.16, the KorAHF registry [10], and CHECK-HF [11], and comparable to the observational cohort by Barry et al. [34]. Despite concerns regarding lower heart rates in the oldest-old, our results indicate that age alone should not preclude beta-blocker therapy when supported by proactive careful monitoring in a specialised heart failure clinic.

Overall, the increasing burden of comorbidities among the oldest-old group may hamper the implementation of treatments [43]. Other potential unexplored reasons to underdosing of HF GDMT pillars were lower socio-economic-education status frailty and polypharmacy [43].

### NYHA assessment, LVEF and clinical outcomes

This study demonstrated that although LVEF improved modestly after 6 months of HF clinic follow-up across all age groups, improvement in NYHA functional class was mainly observed in the youngest-old and middle-old, while the oldest-old showed minimal functional gain despite evidence of cardiac recovery. This dissociation is supported by prior studies, including PROVE-HF, where older patients (≥ 75 years) exhibited comparable reverse remodeling but less improvement in health status, suggesting attenuated symptomatic benefit with aging [44]. In contrast, study in Spain reported significant NYHA improvement in patients aged > 80 years after one year of specialised HF clinic follow-up, suggesting that longer follow-up or differing patient profiles may influence functional outcomes [45]. The limited functional improvement in the oldest-old likely reflects the multifactorial nature of symptoms in advanced age. While GDMT promotes ventricular remodeling, functional status is heavily influenced by frailty, sarcopenia, and multimorbidity, which are prevalent in this group and independently associated with adverse outcomes [46]. These factors, together with polypharmacy and treatment limitations, may constrain symptomatic recovery despite structural improvement [47, 48].

In comparison to international data, our cohort demonstrated a distinct pattern of clinical outcomes among older adults with heart failure. The Euro Heart Failure Survey I reported higher mortality in octogenarians (11.7%), nearly double that of younger patients (6.1%), whereas our study observed numerically higher mortality in the youngest-old at 3 and 6 months, though not statistically significant [21]. Similarly, the KorAHF registry showed that HF GDMT pillars improved survival across older age groups, with attenuated benefit in those aged ≥ 80 years [11]. In our cohort, higher mortality in the youngest-old despite greater LVEF improvement may reflect a higher burden of ischemic heart disease and HFrEF, where myocardial recovery is more responsive to therapy, but underlying atherosclerosis and comorbidities drive adverse outcomes. Conversely, the oldest-old showed a trend toward higher readmission despite lower mortality, suggesting a phenotype of frailty, multimorbidity, and recurrent decompensation [46, 49]. This is supported by our regression analysis, where CKD, IHD, and LVEF were not independently associated with outcomes, implying a greater role of non-cardiac factors. Hypertension was associated with increased 6-month readmission, highlighting the impact of comorbidity burden. The lack of association between age group and GDMT prescription further suggests that treatment access alone does not explain outcome differences, and that heterogeneity in disease phenotype and physiological reserve likely underpins these findings.

### Limitations and future directions

Our study’s observational design and lack of a control group limit the ability to draw causal conclusions. Additionally, the absence of digital data hindered data collection and resulted in missing information. A significant number of patients were also lost to follow-up. We may have underestimated the true situation due to the lack of specific cognitive assessments and the absence of records frailty. Frequent cardiology follow-up may have influenced outcomes, while selection bias toward more robust elderly patients in HF clinics—compared to frailer patients managed in geriatric settings—may partly explain the high GDMT implementation. Further research with larger cohorts and randomized trials targeting the oldest-old population is needed to identify more effective interventions for this vulnerable group.

## Conclusion

Despite a higher comorbidity burden and heart failure readmission in the oldest-old, and paradoxically higher mortality in the youngest-old with greater coronary artery disease despite higher HF GDMT use, our study demonstrates that both functional and structural recovery can be achieved consistently across age groups.

These findings indicate that age alone should not preclude optimisation of GDMT, and that differences in outcomes are more likely driven by underlying disease phenotype and comorbidity burden rather than age itself. This highlights the importance of individualised, multidisciplinary care in older adults with heart failure, particularly in addressing frailty, multimorbidity, and treatment tolerance.

Further studies with larger cohorts and prospective or randomised designs are required to better define the risks and benefits of HF GDMT in the oldest-old population. Future research should incorporate assessment of adverse effects, treatment tolerability, frailty, quality of life, and medication adherence, which are critical in determining real-world effectiveness. Such data would support refinement of clinical guidelines and enable more personalised therapeutic strategies to improve long-term outcomes in this growing and vulnerable population. A multidisciplinary approach involving cardiologists, geriatricians, and primary care physicians remains essential to holistically address the complex needs of older patients.

## Supplementary Information

Supplementary Material 1.

Supplementary Material 2.

## Data Availability

Availability of data and materials: The datasets generated and/or analysed during the current study are not publicly available due to institutional regulations and the need to protect patient confidentiality but are available from the corresponding author on reasonable request.

## References

[1] ESCAP. Malaysia | Demographic Changes: population-trends-asiapacific.org. 2024 [Available from: https://www.population-trends-asiapacific.org/data/MYS

[2] Cherubini A, Oristrell J, Pla X, Ruggiero C, Ferretti R, Diestre G, et al. The persistent exclusion of older patients from ongoing clinical trials regarding heart failure. Arch Intern Med. 2011;171(6):550–6. 21444844 10.1001/archinternmed.2011.31

[3] Yusuf S, Pitt B, Davis CE, Hood WB Jr., Cohn JN. Effect of enalapril on mortality and the development of heart failure in asymptomatic patients with reduced left ventricular ejection fractions. N Engl J Med. 1992;327(10):685–91. 1463530 10.1056/NEJM199209033271003

[4] Pitt B, Zannad F, Remme WJ, Cody R, Castaigne A, Perez A, et al. The effect of spironolactone on morbidity and mortality in patients with severe heart failure. Randomized Aldactone Evaluation Study Investigators. N Engl J Med. 1999;341(10):709–17. 10471456 10.1056/NEJM199909023411001

[5] Packer M, Coats AJ, Fowler MB, Katus HA, Krum H, Mohacsi P, et al. Effect of carvedilol on survival in severe chronic heart failure. N Engl J Med. 2001;344(22):1651–8. 11386263 10.1056/NEJM200105313442201

[6] Packer M, Anker SD, Butler J, Filippatos G, Pocock SJ, Carson P, et al. Cardiovascular and Renal Outcomes with Empagliflozin in Heart Failure. N Engl J Med. 2020;383(15):1413–24. 32865377 10.1056/NEJMoa2022190

[7] Ling HS, Chung BK, Chua PF, Gan KX, Ho WL, Ong EYL, et al. Acute decompensated heart failure in a non cardiology tertiary referral centre, Sarawak General Hospital (SGH-HF). BMC Cardiovasc Disord. 2020;20(1):511. 33287705 10.1186/s12872-020-01793-7 PMC7720602

[8] Mohd Ghazi A, Teoh CK, Abdul Rahim AA. Patient profiles on outcomes in patients hospitalized for heart failure: a 10-year history of the Malaysian population. ESC Heart Fail. 2022;9(4):2664–75. 35652407 10.1002/ehf2.13992 PMC9288813

[9] Wan Ahmad WA, Abdul Ghapar AK, Zainal Abidin HA, Karthikesan D, Ross NT, MA SKAK, et al. Characteristics of patients admitted with heart failure: Insights from the first Malaysian Heart Failure Registry. ESC Heart Fail. 2024;11(2):727–36. 38131217 10.1002/ehf2.14608 PMC10966232

[10] Veenis JF, Brunner-La Rocca HP, Linssen GC, Geerlings PR, Van Gent MW, Aksoy I, et al. Age differences in contemporary treatment of patients with chronic heart failure and reduced ejection fraction. Eur J Prev Cardiol. 2019;26(13):1399–407. 30866680 10.1177/2047487319835042 PMC6696738

[11] Seo WW, Park JJ, Park HA, Cho HJ, Lee HY, Kim KH, et al. Guideline-directed medical therapy in elderly patients with heart failure with reduced ejection fraction: a cohort study. BMJ Open. 2020;10(2):e030514. 32034017 10.1136/bmjopen-2019-030514 PMC7044987

[12] Verulava T, Jorbenadze R, Lordkipanidze A, Gongadze A, Tsverava M, Donjashvili M. Readmission after hospitalization for heart failure in elderly patients in Chapidze Emergency Cardiology Center, Georgia. J Health Res. 2021;36(3):575–83.

[13] Murad K, Goff DC Jr., Morgan TM, Burke GL, Bartz TM, Kizer JR, et al. Burden of Comorbidities and Functional and Cognitive Impairments in Elderly Patients at the Initial Diagnosis of Heart Failure and Their Impact on Total Mortality: The Cardiovascular Health Study. JACC Heart Fail. 2015;3(7):542–50. 26160370 10.1016/j.jchf.2015.03.004 PMC4499113

[14] Elam C, Aagaard P, Slinde F, Svantesson U, Hulthén L, Magnusson PS, et al. The effects of ageing on functional capacity and stretch-shortening cycle muscle power. J Phys Ther Sci. 2021;33(3):250–60. 33814713 10.1589/jpts.33.250 PMC8012187

[15] Group KDIGOKCW. KDIGO 2012 Clinical Practice Guideline for the Evaluation and Management of Chronic Kidney Disease. 2013. 10.1038/ki.2013.243 23989362

[16] Ministry of Health Malaysia. Clinical Practice Guidelines: Heart Failure. Putrajaya; 2023.

[17] Albalushi MA, Neshat-Mokadem L, Al-Mawaali G. Evaluation of prescribing adherence to guideline-directed medical therapy in patients with chronic heart failure: a retrospective study at the National Heart Centre in Oman. BMC Cardiovasc Disord. 2025;25(1):762. 41136931 10.1186/s12872-025-05232-3 PMC12551167

[18] Wahab MSA, Zaini MH, Ali AA, Sahudin S, Mehat MZ, Hamid HA, et al. The use of herbal and dietary supplement among community-dwelling elderly in a suburban town of Malaysia. BMC Complement Med Ther. 2021;21(1):110. 33794868 10.1186/s12906-021-03287-1 PMC8017757

[19] Wu JR, Moser DK, DeWalt DA, Rayens MK, Dracup K. Health Literacy Mediates the Relationship Between Age and Health Outcomes in Patients With Heart Failure. Circ Heart Fail. 2016;9(1):e002250. 26721913 10.1161/CIRCHEARTFAILURE.115.002250 PMC4698978

[20] Abdullah JM, Ismail A, Yusoff MSB. Healthy Ageing in Malaysia by 2030: Needs, Challenges and Future Directions. Malays J Med Sci. 2024;31(4):1–13. 39247109 10.21315/mjms2024.31.4.1 PMC11376998

[21] Komajda M, Hanon O, Hochadel M, Follath F, Swedberg K, Gitt A, et al. Management of octogenarians hospitalized for heart failure in Euro Heart Failure Survey I. Eur Heart J. 2007;28(11):1310–8. 17185303 10.1093/eurheartj/ehl443

[22] Benjamin EJ, Blaha MJ, Chiuve SE, Cushman M, Das SR, Deo R, et al. Heart Disease and Stroke Statistics-2017 Update: A Report From the American Heart Association. Circulation. 2017;135(10):e146–603. 28122885 10.1161/CIR.0000000000000485 PMC5408160

[23] Vasan RS, Larson MG, Benjamin EJ, Evans JC, Reiss CK, Levy D. Congestive heart failure in subjects with normal versus reduced left ventricular ejection fraction: prevalence and mortality in a population-based cohort. J Am Coll Cardiol. 1999;33(7):1948–55. 10362198 10.1016/s0735-1097(99)00118-7

[24] Topol EJ, Traill TA, Fortuin NJ. Hypertensive hypertrophic cardiomyopathy of the elderly. N Engl J Med. 1985;312(5):277–83. 2857050 10.1056/NEJM198501313120504

[25] Gerdts E, Zabalgoitia M, Björnstad H, Svendsen TL, Devereux RB. Gender differences in systolic left ventricular function in hypertensive patients with electrocardiographic left ventricular hypertrophy (the LIFE study). Am J Cardiol. 2001;87(8):980-3; a4. 10.1016/s0002-9149(01)01433-3 11305990

[26] Yip GW, Ho PP, Woo KS, Sanderson JE. Comparison of frequencies of left ventricular systolic and diastolic heart failure in Chinese living in Hong Kong. Am J Cardiol. 1999;84(5):563–7. 10482156 10.1016/s0002-9149(99)00378-1

[27] Tso DK, Moe G. Cardiovascular disease in Chinese Canadians: a case-mix study from an urban tertiary care cardiology clinic. Can J Cardiol. 2002;18(8):861–9. 12215749

[28] Sanderson JE, Tse TF. Heart failure: a global disease requiring a global response. Heart. 2003;89(6):585–6. 12748201 10.1136/heart.89.6.585 PMC1767689

[29] Nicholls MG, Richards AM. Is hypertension a leading cause of heart failure in Chinese? Clin Exp Pharmacol Physiol. 2002;29(9):850–1. 12165054 10.1046/j.1440-1681.2002.03735.x

[30] Smilowitz NR, Mahajan AM, Roe MT, Hellkamp AS, Chiswell K, Gulati M, et al. Mortality of Myocardial Infarction by Sex, Age, and Obstructive Coronary Artery Disease Status in the ACTION Registry-GWTG (Acute Coronary Treatment and Intervention Outcomes Network Registry-Get With the Guidelines). Circ Cardiovasc Qual Outcomes. 2017;10(12):e003443. 29246884 10.1161/CIRCOUTCOMES.116.003443

[31] Khoja A, Andraweera PH, Lassi ZS, Ali A, Zheng M, Pathirana MM, et al. Risk Factors for Early-Onset Versus Late-Onset Coronary Heart Disease (CHD): Systematic Review and Meta-Analysis. Heart Lung Circ. 2023;32(11):1277–311. 37777398 10.1016/j.hlc.2023.07.010

[32] McDonagh TA, Metra M, Adamo M, Gardner RS, Baumbach A, Böhm M, et al. 2021 ESC Guidelines for the diagnosis and treatment of acute and chronic heart failure. Eur Heart J. 2021;42(36):3599–726. 34447992 10.1093/eurheartj/ehab368

[33] Heidenreich PA, Bozkurt B, Aguilar D, Allen LA, Byun JJ, Colvin MM, et al. 2022 AHA/ACC/HFSA Guideline for the Management of Heart Failure: A Report of the American College of Cardiology/American Heart Association Joint Committee on Clinical Practice Guidelines. J Am Coll Cardiol. 2022;79(17):e263–421. 35379503 10.1016/j.jacc.2021.12.012

[34] Barry AR, Grewal M, Blain L. Use of Guideline-Directed Medical Therapy in Patients Aged 80 Years or Older With Heart Failure With Reduced Ejection Fraction. CJC Open. 2023;5(4):303–9. 37124968 10.1016/j.cjco.2023.01.002 PMC10140749

[35] Stefil M, Manzano L, Montero-PéRez-Barquero M, Coats AJS, Flather M. New horizons in management of heart failure in older patients. Age Ageing. 2019;49(1):16–9. 31697342 10.1093/ageing/afz122

[36] Gorodeski EZ, Goyal P, Hummel SL, Krishnaswami A, Goodlin SJ, Hart LL, et al. Domain Management Approach to Heart Failure in the Geriatric Patient: Present and Future. J Am Coll Cardiol. 2018;71(17):1921–36. 29699619 10.1016/j.jacc.2018.02.059 PMC7304050

[37] Goyal P, Chen L, Lau JD, Rosenson RS, Levitan EB. Reductions in renin-angiotensin system inhibitors following hospitalization for heart failure. ESC Heart Fail. 2024;11(6):3862–71. 39030944 10.1002/ehf2.14953 PMC11631335

[38] VafeidouK, Psoma O, Apostolidis E, Sarvani A, Doumas M, Kotsa K et al. Clinical Inertia in SGLT2 Inhibitor Use Among Elderly Patients with Type 2 Diabetes and Chronic Kidney Disease: A Comparison of Regional and University Hospital Practice. Geriatr (Basel). 2025;10(6):144. 10.3390/geriatrics10060144. 10.3390/geriatrics10060144 PMC12641619 41283455

[39] Madero M, Chertow GM, Mark PB. SGLT2 Inhibitor Use in Chronic Kidney Disease: Supporting Cardiovascular, Kidney, and Metabolic Health. Kidney Med. 2024;6(8):100851. 39822934 10.1016/j.xkme.2024.100851 PMC11738012

[40] Solomon SD, McMurray JJV, Vaduganathan M, Claggett B, Jhund PS, Desai AS, et al. Finerenone in Heart Failure with Mildly Reduced or Preserved Ejection Fraction. N Engl J Med. 2024;391(16):1475–85. 39225278 10.1056/NEJMoa2407107

[41] Hobbs FDR, McManus RJ, Taylor CJ, Jones NR, Rahman JK, Wolstenholme J, et al. Low-dose spironolactone and cardiovascular outcomes in moderate stage chronic kidney disease: a randomized controlled trial. Nat Med. 2024;30(12):3634–45. 39349629 10.1038/s41591-024-03263-5 PMC11753262

[42] Bhandari S, Mehta S, Khwaja A, Cleland JGF, Ives N, Brettell E, et al. Renin-Angiotensin System Inhibition in Advanced Chronic Kidney Disease. N Engl J Med. 2022;387(22):2021–32. 36326117 10.1056/NEJMoa2210639

[43] Stolfo D, Sinagra G, Savarese G. Evidence-based Therapy in Older Patients with Heart Failure with Reduced Ejection Fraction. Card Fail Rev. 2022;8:e16. 35541287 10.15420/cfr.2021.34 PMC9069263

[44] Murphy SP, Ward JH, Piña IL, Felker GM, Butler J, Maisel AS, et al. Age Differences in Effects of Sacubitril/Valsartan on Cardiac Remodeling, Biomarkers, and Health Status. JACC Heart Fail. 2022;10(12):976–88. 36456072 10.1016/j.jchf.2022.07.001

[45] AraújoCS, Marco I, Restrepo-Córdoba MA, Vila Costa I, Pérez-Villacastín J, Goirigolzarri-Artaza J. An Observational Study of Evidence-Based Therapies in Older Patients with Heart Failure with Reduced Ejection Fraction: Insights from a Dedicated Heart Failure Clinic. J Clin Med. 2024;13(23):7171. 10.3390/jcm13237171. 10.3390/jcm13237171 PMC11642353 39685630

[46] Wu JR, Moser DK. Medication Adherence Mediates the Relationship Between Heart Failure Symptoms and Cardiac Event-Free Survival in Patients With Heart Failure. J Cardiovasc Nurs. 2018;33(1):40–6. 28591004 10.1097/JCN.0000000000000427 PMC5714687

[47] Talha KM, Pandey A, Fudim M, Butler J, Anker SD, Khan MS. Frailty and heart failure: State-of-the-art review. J Cachexia Sarcopenia Muscle. 2023;14(5):1959–72. 37586848 10.1002/jcsm.13306 PMC10570089

[48] Salmon T, Essa H, Tajik B, Isanejad M, Akpan A, Sankaranarayanan R. The Impact of Frailty and Comorbidities on Heart Failure Outcomes. Card Fail Rev. 2022;8:e07. 35399550 10.15420/cfr.2021.29 PMC8977991

[49] DenfeldQE, Jha SR, Fung E, Jaarsma T, Maurer MS, Reeves GR, Afilalo J, Beerli N, Bellumkonda L, De Geest S, Gorodeski EZ, Joyce E, Kobashigawa J, Mauthner O, McDonagh J, Uchmanowicz I, Dickson VV, Lindenfeld J, Macdonald P. Assessing and managing frailty in advanced heart failure: An International Society for Heart and Lung Transplantation consensus statement. J Heart Lung Transplant. 2023:S1053-2498(23)02028-4. 10.1016/j.healun.2023.09.013. 10.1016/j.healun.2023.09.013 38099896

