# Supplementary material for: Guideline-directed medical therapy in older adults with heart failure; Are there differences across age group?
Source: BMC Geriatr. 2026 May 7;26:856. doi: 10.1186/s12877-026-07571-y (PMC13281586; doi:10.1186/s12877-026-07571-y)
Supplement: Supplementary file 1 — Supplementary Material 1. [file 12877_2026_7571_MOESM1_ESM.docx]

Supplementary Fig. 2a: RASi prescriptions across age groups at baseline, 3-month and 6-month


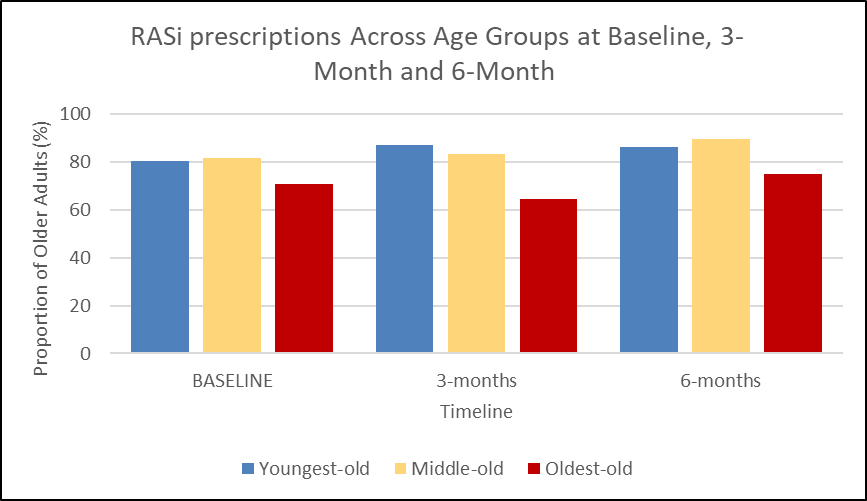


p=0.40

p=0.23

*p* = 0.387

*p* = 0.226

*p* = 0.020*

Supplementary Fig. 2b: BB prescriptions across age groups at baseline, 3-month and 6-month


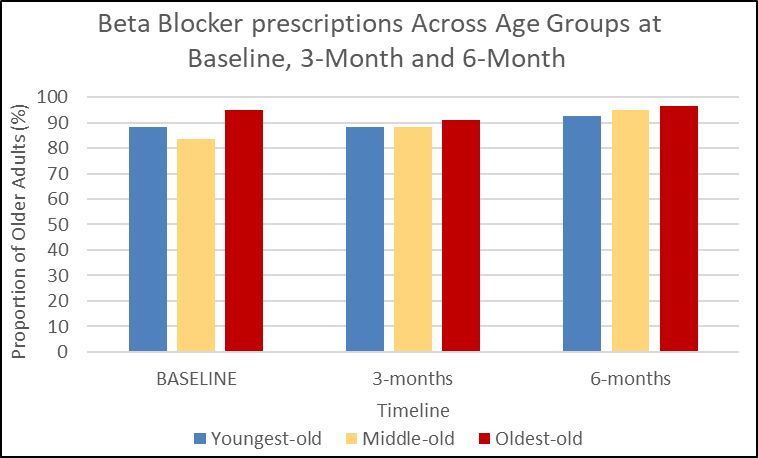


p=0.25

p=0.89

p=0.89

*p* = 0.728

*p* = 0.613

*p* = 0.233

Supplementary Fig. 2c: MRA prescriptions across age groups at baseline, 3-month and 6-month


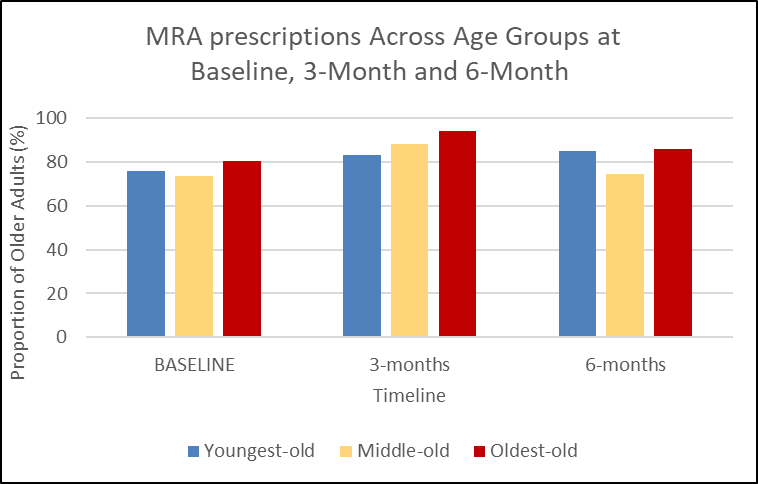


p=0.74

p=0.26

p=0.37

*p* = 0.711

*p* = 0.821

*p* = 0.735

Supplementary Fig. 2d: SGLT2i prescriptions across age groups at baseline, 3-month and 6-month


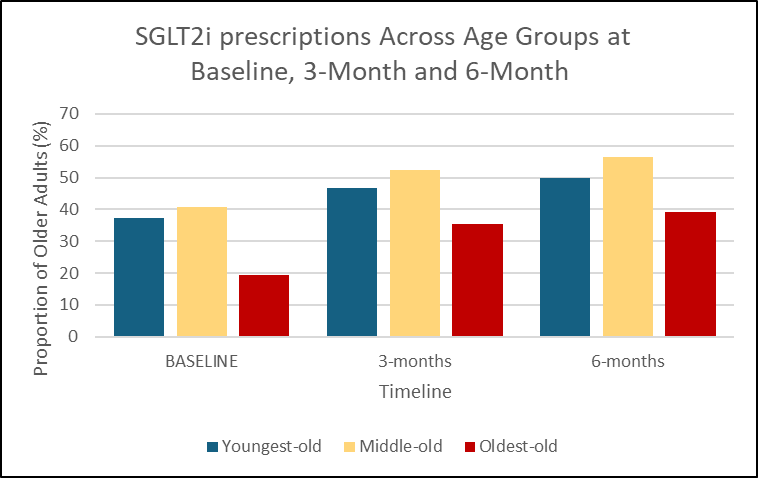


p=0.08

p=0.33

p=0.38

*p* = 0.137

*p* = 0.240

*p* = 0.805

Fig: Figure; RASi: Renin Angiotensin System inhibitor; BB: Beta Blocker; MRA: Mineralocorticoid Antagonist; SGLT2i: Sodium-Glucose Cotransporter inhibitor. *p value is significant.
