# Supplementary material for: Guideline-directed medical therapy in older adults with heart failure; Are there differences across age group?
Source: BMC Geriatr. 2026 May 7;26:856. doi: 10.1186/s12877-026-07571-y (PMC13281586; doi:10.1186/s12877-026-07571-y)
Supplement: Supplementary file 2 — Supplementary Material 2. [file 12877_2026_7571_MOESM2_ESM.docx]

Supplementary Table 1: Number of HF GDMT pillars across age groups at baseline, 3-month and 6-month

|  |  | Age group (n=176) | | | | | |  |
| --- | --- | --- | --- | --- | --- | --- | --- | --- |
|  |  | 65-70  Youngest-old  (n=86) | | 71-75  Middle-old  (n=49) | | >75  Oldest-old  (n=41) | |  |
|  |  |  |  |  |  |  |  |  |
|  |  |  |  |  |  |  |  |  |
| Month | No. of HF GDMT Pillar | n | (%) | n | (%) | n | (%) | *p* value |
| 0 | 0 | 1 | (1.2) | 0 | (0) | 0 | (0) | 0.093 |
|  | 1 | 7 | (8.1) | 6 | (12.2) | 1 | (2.4) |  |
|  | 2 | 21 | (24.4) | 10 | (20.4) | 15 | (36.6) |  |
|  | 3 | 35 | (40.7) | 21 | (42.9) | 22 | (53.7) |  |
|  | 4 | 22 | (25.6) | 12 | (24.5) | 3 | (7.3) |  |
| 3 | 0 | 0 | (0) | 0 | (0) | 0 | (0) | 0.261 |
|  | 1 | 8 | (10.4) | 2 | (4.8) | 2 | (5.9) |  |
|  | 2 | 9 | (11.7) | 9 | (21.4) | 9 | (26.5) |  |
|  | 3 | 31 | (40.3) | 13 | (31.0) | 15 | (44.1) |  |
|  | 4 | 29 | (37.7) | 18 | (42.9) | 8 | (23.5) |  |
| 6 | 0 | 0 | (0) | 0 | (0) | 0 | (0) | 0.587 |
|  | 1 | 3 | (4.5) | 2 | (5.1) | 1 | (3.6) |  |
|  | 2 | 13 | (19.7) | 8 | (20.5) | 6 | (21.4) |  |
|  | 3 | 22 | (33.3) | 11 | (28.2) | 14 | (50.0) |  |
|  | 4 | 28 | (42.4) | 18 | (46.2) | 7 | (25.0) |  |

Supplementary Table 2: Older adults achieving at least 50% target dosing of HF GDMT pillars across age groups at 3-month and 6-month

|  |  | Age group (n=176) | | | | | |  |
| --- | --- | --- | --- | --- | --- | --- | --- | --- |
|  |  | 65-70  Youngest-old  (n=86) | | 71-75  Middle-old  (n=49) | | >75  Oldest-old  (n=41) | |  |
|  |  |  |  |  |  |  |  |  |
|  |  |  |  |  |  |  |  |  |
| Drug | Month | n | (%) | n | (%) | n | (%) | *p* value |
| RASi | 3 | 45 | (41.6) | 22 | (52.4) | 15 | (18.3) | 0.371 |
|  | 6 | 37 | (56.1) | 25 | (64.1) | 12 | (42.9) | 0.233 |
| BB | 3 | 37 | (50.7) | 17 | (40.5) | 19 | (57.6) | 0.322 |
|  | 6 | 37 | (58.7) | 16 | (41.0) | 16 | (59.3) | 0.191 |
| MRA | 3 | 46 | (62.2) | 25 | (61.0) | 18 | (52.9) | 0.705 |
|  | 6 | 43 | (69.4) | 21 | (61.8) | 16 | (59.3) | 0.598 |

RASi: Renin Angiotensin System inhibitor; BB: Beta Blocker; MRA: Mineralocorticoid Antagonist; SGLT2i: Sodium-Glucose Cotransporter inhibitor. *p value is significant.

Supplementary Table 3: NYHA functional class across age groups at baseline, 3-month, 6-month

|  |  | Age group (n=176) | | | | | |  |
| --- | --- | --- | --- | --- | --- | --- | --- | --- |
|  |  | 65-70  Youngest-old  (n=86) | | 71-75  Middle-old  (n=49) | | >75  Oldest-old  (n=41) | |  |
|  |  |  |  |  |  |  |  |  |
|  |  |  |  |  |  |  |  |  |
| Month | NYHA | n | (%) | n | (%) | n | (%) | *p* value |
| 0 | 1 | 21 | (26.9) | 8 | (18.6) | 4 | (13.8) | 0.325 |
|  | 2 | 43 | (55.1) | 22 | (51.2) | 17 | (58.6) |  |
|  | 3 | 12 | (15.4) | 13 | (30.2) | 8 | (27.6) |  |
|  | 4 | 2 | (2.6) | 0 | (0) | 0 | (0) |  |
| 3 | 1 | 33 | (52.4) | 13 | (44.8) | 7 | (30.4) | 0.460 |
|  | 2 | 25 | (39.7) | 13 | (44.8) | 13 | (56.5) |  |
|  | 3 | 5 | (7.9) | 3 | (10.3) | 3 | (13.0) |  |
|  | 4 | 0 | (0) | 0 | (0) | 0 | (0) |  |
| 6 | 1 | 28 | (58.3) | 9 | (40.9) | 7 | (36.8) | 0.055 |
|  | 2 | 15 | (31.3) | 13 | (59.1) | 8 | (42.1) |  |
|  | 3 | 5 | (10.4) | 0 | (0) | 4 | (21.1) |  |
|  | 4 | 0 | (0) | 0 | (0) | 0 | (0) |  |

NYHA: New York Heart Association. *p value is significant.

Supplementary Table 4: Mean LV ejection fraction (EF) across age groups at baseline, 3-month and 6-month

|  | Age group (n=176) | | | | | | | | |  |
| --- | --- | --- | --- | --- | --- | --- | --- | --- | --- | --- |
|  | 65-70  Youngest-old  (n=86) | | | 71-75  Middle-old  (n=49) | | | >75  Oldest-old  (n=41) | | |  |
|  |  |  |  |  |  |  |  |  |  |  |
|  |  |  |  |  |  |  |  |  |  |  |
| Month | Mean LVEF, % | | SD | Mean LVEF, % | | SD | Mean LVEF, % | SD | | *p* value |
| 0 | 29.0 | | 9.7 | 35.5 | | 14.7 | 36.2 | 12.3 | | 0.001* |
| 3 | 30.1 | | 11.9 | 38.3 | | 8.14 | 43.8 | 20.7 | | 0.141 |
| 6 | 41.5 | | 17.3 | 38.5 | | 10.2 | 40.2 | 17.5 | | 0.893 |
| Bonferroni post hoc analysis at baseline (Month 0) | | Age group | | | Mean Difference | | | | *p* value | |
|  |  | 65-70 vs 71-75 | | | -6.56 | | | | 0.008* | |
|  |  | 65-70 vs >75 | | | -7.21 | | | | 0.006* | |
|  |  | 71-75 vs >75 | | | -0.66 | | | | 1.0 | |

SD: Standard Deviation. *p value is significant.

Supplementary Table 5: Multivariate logistic regression of factors associated with HF GDMT pillars prescription and clinical outcomes at 3-month and 6-month

| All-cause mortality at 3-month | | | |
| --- | --- | --- | --- |
| Variable | Adjusted OR | 95% CI | *p* value |
| Age group (Middle-old) | 1.49 | 0.23-9.34 | 0.674 |
| Age group (Oldest-old) | 0.39 | 0.03-4.9 | 0.470 |
| Gender | 1.21 | 0.25-5.75 | 0.807 |
| Hypertension | 2.96 | 0.70-12.4 | 0.137 |
| DM | 0.27 | 0.05-1.25 | 0.095 |
| CKD | 1.68 | 0.29-9.61 | 0.559 |
| IHD | 1.36 | 0.35-5.32 | 0.653 |
| LVEF | 1.02 | 0.6-1.09 | 0.461 |
| All-cause mortality at 6-month | | | |
| Age group (Middle-old) | 1.36 | 0.34-5.39 | 0.654 |
| Age group (Oldest-old) | 0.72 | 0.15-3.43 | 0.689 |
| Gender | 0.50 | 0.17-1.50 | 0.221 |
| Hypertension | 3.06 | 0.95-9.80 | 0.059 |
| DM | 0.38 | 0.12-1.23 | 0.109 |
| CKD | 1.04 | 0.31-3.48 | 0.943 |
| IHD | 1.33 | 0.46-3.81 | 0.589 |
| LVEF | 1.02 | 0.98-1.07 | 0.295 |
| HF Readmission at 3-month | | | |
| Age group (Middle-old) | 0.60 | 0.113.25 | 0.556 |
| Age group (Oldest-old) | 0.74 | 0.13-4.23 | 0.743 |
| Gender | 0.64 | 0.16-2.46 | 0.517 |
| Hypertension | 3.00 | 0.71-12.56 | 0.132 |
| DM | 0.40 | 0.09-1.70 | 0.220 |
| CKD | 1.56 | 0.29-8.46 | 0.601 |
| IHD | 0.69 | 0.17-2.70 | 0.597 |
| LVEF | 1.00 | 0.94-1.06 | 0.859 |
| HF Readmission at 6-month | | | |
| Age group (Middle-old) | 0.51 | 0.09-2.76 | 0.440 |
| Age group (Oldest-old) | 1.12 | 0.23-5.53 | 0.881 |
| Gender | 0.50 | 0.14-1.76 | 0.286 |
| Hypertension | 4.10 | 1.00-16.81 | 0.049* |
| DM | 0.40 | 0.09-1.65 | 0.208 |
| CKD | 1.17 | 0.27-5.09 | 0.831 |
| IHD | 0.60 | 0.16-2.26 | 0.453 |
| LVEF | 0.99 | 0.94-1.050 | 0.891 |
| HF GDMT pillars prescription (No. of types) at 3-month | | | |
| Age group (Middle-old) | 1.20 | 0.52 – 2.80 | 0.668 |
| Age group (Oldest-old) | 1.58 | 0.65 – 3.85 | 0.312 |
| Gender | 2.32 | 1.17 – 4.60 | 0.016* |
| Hypertension | 0.59 | 0.28 – 1.23 | 0.160 |
| DM | 0.48 | 0.25 – 0.94 | 0.031* |
| CKD | 1.95 | 0.94 – 4.03 | 0.073 |
| IHD | 1.14 | 0.60 – 2.14 | 0.690 |
| LVEF | 0.996 | 0.97 – 1.01 | 0.801 |
| HF GDMT pillars prescription (No. of types) at 6-month | | | |
| Age group (Middle-old) | 1.01 | **0.40 – 2.52** | **0.990** |
| Age group (Oldest-old) | 1.60 | **0.61 – 4.18** | **0.337** |
| Gender | 2.73 | **1.26 – 5.93** | **0.011*** |
| Hypertension | 0.61 | **0.27 – 1.42** | **0.251** |
| DM | 0.48 | **0.23 – 1.00** | **0.051** |
| CKD | 1.44 | **0.64 – 3.23** | **0.377** |
| IHD | 1.62 | **0.81 – 3.23** | **0.175** |
| LVEF | 0.97 | **0.94 – 1.01** | **0.159** |

OR: Odds Ratio; 95% CI: 95% Confidence Interval. *p value is significant.
